# Summary of the National Advisory Committee on Immunization (NACI) statement on the prevention of respiratory syncytial virus (RSV) in older adults

**DOI:** 10.14745/ccdr.v51i08a01

**Published:** 2025-08-28

**Authors:** April Killikelly, Winnie Siu, Elissa M Abrams, Nicholas Brousseau

**Affiliations:** 1 Public Health Agency of Canada, Centre for Immunization and Respiratory Infectious Diseases, Ottawa, ON; 2 School of Epidemiology and Public Health, Faculty of Medicine, University of Ottawa, Ottawa, ON; 3 Department of Pediatrics and Child Health, Department of Pediatrics; Section of Allergy and Immunology, University of Manitoba, Winnipeg, MB; 4 Department of Pediatrics, Division of Allergy, University of British Columbia, Vancouver, BC; 5 Département de médecine sociale et préventive, Faculté de médecine, Université Laval, Québec, QC

**Keywords:** National Advisory Committee on Immunization, RSV, older adults, RSVPreF3/Arexvy, RSVpreF/Abrysvo, mRNA-1345/mRESVIA, vaccine

## Abstract

**Background:**

Respiratory syncytial virus (RSV) is a common respiratory virus. In addition to infants, older adults are at higher risk of severe outcomes due to RSV, particularly advanced-age older adults and those with chronic medical conditions. The authorization of three vaccines, one for adults 50 years of age and older (Arexvy) and two for adults 60 years of age and older (Abrysvo and mRESVIA), offers the opportunity to protect older Canadians from RSV disease. This article summarizes guidance from the National Advisory Committee on Immunization (NACI) on the prevention of RSV in older adults.

**Methods:**

NACI established key policy questions and performed an evidence review and synthesis for three new vaccines. In consideration of the burden of illness to be prevented, safety and efficacy of the new immunizing products, economic evidence and ethics, equity, feasibility and acceptability considerations, NACI made evidence-based recommendations.

**Results:**

The three RSV vaccines may provide similar reductions in hospitalizations associated with RSV and medically attended RSV respiratory tract infection for adults 60 years of age and older. However, evidence is limited for other outcomes. These vaccines were well-tolerated in clinical studies, with an acceptable safety profile among older adults. The duration of protection of the RSV vaccine is not yet known, and it is unclear if the protection offered by vaccination can be boosted by subsequent doses of vaccine.

**Conclusion:**

Based on available evidence, NACI recommends RSV immunization programs for adults 75 years of age and older, particularly for older adults with chronic health conditions who are at increased risk of severe RSV disease. NACI also recommends RSV immunization programs for adults 60 years of age and older who are residents of nursing homes and other chronic care facilities. NACI recommends that receiving an RSV vaccine may be considered as an individual decision by adults 50 to 74 years of age, in consultation with their healthcare provider.

## Introduction

Respiratory syncytial virus (RSV) is a common respiratory virus. In addition to infants, older adults, particularly advanced-age older adults and those with chronic medical conditions, such as cardiopulmonary disease and immunocompromise, are at higher risk of severe outcomes due to RSV ((1)). Patients who reside in chronic care facilities and are admitted to hospital also have a higher likelihood of severe clinical outcomes, including death, compared to patients with other living situations at hospital admission. Primary infection does not confer protective immunity against reinfections, which recur throughout life and become more serious with advanced age in older adults. In addition, adults may be at increased risk of severe RSV disease due to factors that intersect with social determinants of health.

Respiratory syncytial virus has a seasonal pattern of activity, where infections are usually more common in the winter with variation in the timing and magnitude of the peak. Prior to the COVID-19 pandemic, the RSV season in most of Canada was typically November to April, but this may vary by region.

Health Canada has recently authorized three immunization products, based on the pre-fusion stabilized F protein (preF) from RSV. An unadjuvanted vaccine, RSVpreF (Abrysvo, Pfizer) is authorized with an indication for all adults 60 years of age and over. This formulation is also authorized for pregnant women and pregnant people who are 32 to 36 weeks gestational age (wGA) to protect infants from RSV. An AS01E adjuvanted vaccine, RSVPreF^3^ (Arexvy, GSK) is authorized with an indication for all adults 60 years of age and over and for adults at high risk for RSV disease who are 50 to 59 years of age. Authorized for use in all adults aged 60 years and older, mRNA-1345 (mRESVIA, Moderna) delivers preF via an mRNA platform.

The National Advisory Committee on Immunization (NACI) provides the Public Health Agency of Canada with recommendations ((2,3)) regarding the use of vaccines and immunization products for RSV, which reflect the latest evidence for RSV epidemiology, clinical outcomes (such as immunogenicity, efficacy and effectiveness, and safety), immunization practices, and product authorization and availability in Canada. Recommendations also take into account ethics, equity, feasibility and acceptability considerations, and economic analysis. Development of this guidance was triggered by the authorization of new vaccines to protect older adults from RSV. This work was led by NACI’s RSV Working Group (WG) and involved a thorough review and evaluation of the literature, as well as discussion and debate at the scientific and clinical practice levels.

## Methods

The NACI RSV WG reviewed key questions and performed evidence reviews and syntheses. The WG proposed recommendations for vaccine use to NACI in consideration of the burden of illness to be prevented, safety, efficacy, ethics, equity, acceptability, feasibility and economics. All evidence was reviewed according to a Grading of Recommendations, Assessment, Development, and Evaluations (GRADE)-informed methodology and summarized in evidence tables. NACI approved specific evidence-based recommendations and summarized the rationale and relevant considerations in a statement.

## Results

### Efficacy

The evidence suggests that using RSVpreF, RSVPreF3 and mRNA-1345 may result in similar reductions in hospitalizations associated with RSV and medically attended RSV respiratory tract infection (RTI) for adults 60 years of age and older. However, there was limited evidence on the effect of these vaccines against death due to RSV and intensive care unit (ICU) admission associated with RSV. Larger populations may be needed to observe and assess these severe clinical outcomes. As no head-to-head trials currently exist comparing these products, there are important limitations to comparing across RSV vaccine trials for different products due to differences in trial design, including clinical endpoints and follow-up time.

In adults 75 years of age and older, protection against medically attended RSV RTI ranged from roughly 49% to 78% ((4,5)). In adults 75 years of age and older, protection against death, ICU admission and hospitalization was not estimable due to lack of data from clinical trials.

In adults 60 years of age and older, protection against medically attended RSV RTI ranged from roughly 66% to 86%. Data limitations did not allow for estimations of protection against death and ICU for adults 60 years of age and older. Notably, the vaccine trials were conducted during RSV seasons when public health measures due to the COVID-19 pandemic were in effect. These measures reduced the transmission of respiratory viruses, which could explain the low rate of RSV-associated outcomes observed in the trials.

Post-market data have also shown effectiveness of RSVPreF3 and RSVpreF in Phase IV studies. Real-world effectiveness data show that RSV vaccination provides protection against severe RSV disease. Vaccine effectiveness was similar to results from Phase III trials and no substantial differences in effectiveness were observed between products.

The duration of protection provided by the RSV vaccine is still unknown, and it remains unclear whether subsequent doses of vaccine can boost this protection.

## Discussion

### Safety

Respiratory syncytial virus vaccines were well tolerated, with an acceptable safety profile among older adults. In randomized controlled trials, most (greater than 95%) of reported adverse events (AEs) were mild to moderate. The available evidence suggests that RSVpreF may result in a slight increase in severe local AEs and little to no difference in severe systemic AEs compared to placebo. For RSVPreF^3^ and for mRNA-1345, data suggest that vaccination results in a slight increase in severe local and systemic AEs compared to placebo.

Early post-marketing safety data from the United States suggests a potential increased rate of Guillain-Barré syndrome in adults 60 years of age and older after administration of the RSVpreF or RSVPreF^3^ vaccines ((6,7)). However, the currently available preliminary data are subject to limitations. Additional analyses are planned to further assess this potential increased risk of Guillain-Barré syndrome.

### Ethics, equity, feasibility, and acceptability

NACI considered age-based, as well as medical- and social risk-based, RSV vaccine recommendations for older adults. An age-based recommendation would improve both equity and feasibility, as it reduces access barriers, for example, by allowing vaccination in a wider range of settings and making eligibility easier to determine. Furthermore, an age-based recommendation would capture those individuals who have medical conditions that place them at increased risk of severe RSV disease but have not yet been diagnosed. However, equity could also potentially be increased through a risk-based recommendation, given that older adults at greater risk of severe illness would be prioritized.

When interpreting the epidemiological trends to inform the recommendations, equity considerations include acknowledgement that available evidence for some populations is limited and may be biased, for example, due to systemic limitations in available data for racialized groups. Consideration should be made for diverse contexts of equity-implicated communities. One example where diverse contexts may apply is Indigenous groups across various settings (e.g., urban, rural, on-reserve, off-reserve).

NACI acknowledges the feasibility concerns of the different storage temperature for mRNA-1345 and supports jurisdictions in weighing this factor alongside other vaccine characteristics when considering product selection and program design.

### Economics

To support decision-making for the use of vaccines for preventing RSV in adults, NACI conducted a systematic literature review ((8)), developed a de novo model-based economic evaluation ((9)), and performed a multi-model comparison ((10)). The systematic review showed that, in general, without a substantial reduction in vaccine price, the use of RSV vaccines in all adults aged 60 years and older or 65 years and older was unlikely to be cost-effective at commonly used cost-effectiveness thresholds. The model-based economic analysis showed that medical risk-based vaccination strategies could be cost-effective, with the age cutoff for such a policy dependent on model assumptions. Age-based vaccination strategies may offer a positive net health benefit compared to no vaccination; however, they are not resource-efficient compared to medical risk-based strategies. The results of the multi-model comparison were consistent with the de novo model-based economic evaluation. Using currently available vaccine efficacy data, mRNA-1345 may be less cost-effective than other authorized RSV vaccines. If the assumption of lower vaccine efficacy for mRNA-1345 compared to protein subunit vaccines is accurate, a lower vaccine price for mRNA-1345 would reduce the difference in cost-effectiveness. However, true differences in efficacy remain uncertain.

For individuals who may seek vaccination outside of a public health program, NACI recommends that RSV vaccines may be considered as an individual decision by adults 50 to 74 years of age, in consultation with their healthcare provider. It is unknown at this time if these vaccines can be boosted by subsequent doses, and therefore, healthy individuals under 75 years of age may wish to discuss with their healthcare provider whether to defer vaccination until they are at greater risk. If an individual over the age of 75 is not included in a publicly funded program, NACI recommends vaccination for these individuals, particularly for those adults at increased risk of severe RSV disease.

The RSV vaccine is optimally administered just before the start of the RSV season. Jurisdictions are encouraged to define the RSV season and administer RSV vaccines based on local epidemiology (before the COVID-19 pandemic, the RSV season was typically November to April).

### Limitations

Given the need for older adults to be protected from multiple vaccine-preventable diseases, some of which are seasonal, concurrent administration of an RSV vaccine with other adult vaccines is acceptable and supported. If possible, RSV vaccines should be given at least six weeks before or after non-seasonal vaccines (e.g., shingles or diphtheria-tetanus vaccines) to avoid inadvertently attributing an adverse event from another vaccine to the RSV vaccine or vice versa.

### Recommendations

NACI recommends RSV immunization programs for adults 75 years of age and older, particularly for older adults with chronic health conditions who are at increased risk of severe RSV disease. Adults with chronic health conditions, who are at increased medical risk for severe RSV disease, are highlighted in **List 1**. Indigenous Peoples may experience a disproportionate burden of illness due to social, environmental, and economic factors, rooted in the history of colonization and systemic racism (i.e., structural inequity). In First Nations, Métis, and Inuit communities, autonomous decisions should be made by Indigenous Peoples with the support of healthcare and public health partners.

**List 1: Clinically significant chronic health conditions for which respiratory syncytial virus vaccination is particularly important**

**Table:** 

• Cardiac or pulmonary disorders (includes chronic obstructive pulmonary disease, asthma, cystic fibrosis, and conditions affecting ability to clear airway secretions) • Diabetes mellitus and other metabolic diseases • Moderate and severe immunodeficiency (refer to the list of immunocompromising conditions developed for COVID-19) • Chronic renal disease • Chronic liver disease • Neurologic or neurodevelopmental conditions (includes neuromuscular, neurovascular, neurodegenerative [e.g., dementia], neurodevelopmental conditions, and seizure disorders, but excludes migraines and psychiatric conditions without neurological conditions) • Class 3 obesity (defined as BMI of 40 kg/m^2^ and over)

Abbreviation: BMI, body mass index

NACI also recommends RSV immunization programs for adults 60 years of age and older who are residents of nursing homes and other chronic care facilities.

## Conclusion

NACI continues to recommend RSV vaccination for adults 75 years of age and older and adults 60 years of age and older living in long-term care. Respiratory syncytial virus vaccination is particularly recommended for adults at increased risk of RSV disease. NACI will continue to monitor additional evidence, as it emerges, on RSV disease burden and RSV vaccine efficacy and safety in younger age groups.
